# Expanding the dimensions of knowledge hiding: testing a moderated mediation model and analyzing the mediating role of psychological distress using PLS-SEM

**DOI:** 10.3389/fpsyg.2023.1279964

**Published:** 2023-11-27

**Authors:** Xiu Jin, Shanyue Jin, Chenglin Qing

**Affiliations:** ^1^College of Business, Gachon University, Seongnam, Republic of Korea; ^2^Department of Business Administration, Honam University, Gwangju, Republic of Korea

**Keywords:** exploitative leadership, psychological distress, leader’s incivility, knowledge hiding, moderated mediation model, partial least squares structural equation modeling

## Abstract

This study sheds light on the literature on knowledge-hiding behavior in organizations and highlights a better and deeper understanding of the reasons for giving rise to knowledge hiding. In recent decades, knowledge hiding has been subjected to numerous studies in systematic literature reviews and organizational management regarding its impact on outcomes such as individual and organizational performance; however, the mechanism by which knowledge hiding is influenced by antecedents and the process of leading knowledge hiding has not been actively verified. In addition, most previous studies have classified knowledge hiding into one-factor or three-factor dimensions: evasive hiding, playing dumb, and rationalized hiding. To address these issues and limitations, we aimed to conduct empirical research, which have focused on four new dimensions (playing dumb, evasive hiding, rationalized hiding, and procrastination) of knowledge-hiding behavior. Unlike previous research, we provide a research framework for the process of hiding knowledge and verify the significance of the research model, drawing on the social exchange theory and conservation of resources theory to explore and verify the process of hiding knowledge. Specifically, we argue that knowledge hiding is caused by exploitative leadership, and psychological distress as mediators in the relationship between these two variables. Moreover, the moderating and mediating effects of leader incivility were verified. To empirically test the research model, a survey was conducted with 287 employees from small- and medium-sized enterprises in China. Partial least squares structural equation modeling (PLS-SEM), SPSS PROCESS, and AMOS software were used for statistical analyzes. The findings provide evidence that exploitative leadership positively influences both psychological distress and the four dimensions of knowledge hiding. In addition, the mediating effect of psychological distress and the moderating effect of leader incivility were verified and shown to be statistically significant. Based on these findings, the theoretical and practical implications, limitations, and directions for future research are discussed. Overall, the most important contribution is expanding the research field, as this is the first empirical study on the four dimensions of knowledge hiding.

## 1 Introduction

The global business environment has recently become more knowledge-oriented and technology-intensive (Jones and Ratten, 2020; Jafari-Sadeghi et al., 2021, 2022). Business growth usually occurs when knowledge-based organizations combine data, information, and knowledge (Davis and Botkin, 1994). In particular, the importance of the knowledge possessed by organizational members for organizational development and the role of utilizing knowledge have been emphasized. Based on the role of knowledge in facilitating organizational growth, organizations have been demanding that organizational members share their knowledge with colleagues. Organizational members’ knowledge sharing is regarded as an engine that promotes organizational growth, economic growth, and business flourishing (Khoreva and Wechtler, 2020). Most previous studies have demonstrated the role of knowledge sharing and verified the relationship between knowledge sharing and individual and organizational performance (Li et al., 2020; Singh et al., 2021; Olan et al., 2022; Yue et al., 2022; Jia et al., 2023).

Although the extant literature has focused on organizational members’ knowledge-sharing and its driving factors, little research has focused on factors that inhibit individuals’ knowledge-sharing behavior and facilitate them to hide their knowledge (Ghani et al., 2020). Furthermore, few studies have explored knowledge hiding in a culturally diverse workforce (Babic et al., 2018; Miminoshvili and Černe, 2022). Unlike the lack of research exploring the process behind knowledge hiding, studies have been conducted on the negative effects of knowledge hiding. For example, previous research has suggested that knowledge hiding is negatively related to innovative work behavior (Mubarak et al., 2022) and task performance (Singh, 2019). Furthermore, a higher level of knowledge hiding prevents organizational members from delivering creative input, indicating that it is negatively associated with employee creativity (Jahanzeb et al., 2019). These statements suggest that a greater and deeper understanding of both the reason for and the process underlying knowledge hiding in diverse workplaces is urgently needed.

Leadership is a significant antecedent variable that can give rise to knowledge hiding in small- and medium-sized enterprises (SMEs) (Xu et al., 2022). We argue that knowledge hiding is related to exploitative leadership. Exploitative leadership is a destructive leadership pattern (Abdulmuhsin et al., 2021) that utilizes subordinates solely to the leader’s own interests (Feng et al., 2022), and such a style of leadership can be considered egoist (Wu et al., 2021). The characteristics of an exploitative leader are that such leaders are selfish and seek to gain personal benefit by exploiting their subordinates, which impacts employees’ knowledge-hiding behavior (Peng et al., 2019; Abdulmuhsin et al., 2021).

Based on the above, this study aimed to identify the causal relationship between exploitative leadership and knowledge hiding. Moreover, we verified the role of exploitative leadership and its effects on knowledge hiding and predicted that exploitative leadership would lead to knowledge hiding through psychological distress. Rubbab et al. (2022) suggested that an organizational member’s psychological distress plays the role of an explanatory mechanism in the relationship between organizational dehumanization and knowledge hiding. Specifically, dehumanization consumes the psychological resources of organizational members and acts as a stressor by causing psychological distress. This process leads organizational members to use defensive tactics to recover resources or stop their resource loss cycle. Eventually, organizational members’ defensive techniques take the form of knowledge-hiding behavior (Rubbab et al., 2022). We predict that psychological distress is expected to have a mediating role in the effect of exploitative leadership on knowledge hiding. In particular, previous studies have emphasized the mediating effect of psychological distress as the main cause of knowledge hiding. For example, Bhatti et al. (2023) have demonstrated the significant mediating role of psychological distress in the relationship between organizational ostracism and knowledge hiding. Additionally, Rubbab et al. (2022) verified the significant mediating effect of psychological distress in the relationship between organizational dehumanization and knowledge hiding. In particular, the main reasons selecting small and medium-sized enterprises is more suitable for this research are as follows. The concept of exploitative leadership is a newly emerged leadership style. Most studies of exploitative leadership thus far have focused on hospitals (Majeed and Fatima, 2020), hotels (Ye et al., 2022), logistics companies (Lyu et al., 2023), and the education sector (Akhtar et al., 2022).

The characteristic of organizational dehumanization is a type of organizational mistreatment through showing little respect and interest in employees and treating them as a tool to achieve organizational goals (Rubbab et al., 2022). Importantly, there are similar aspects between the characteristics of exploitative leadership and dehumanization. In particular, an exploitative leader is egoistic, inherently self-serving, exerts pressure, manipulates, and treats subordinates as a tool to achieve their own goals (Akhtar et al., 2022; Basaad et al., 2023). Hence, exploitative leadership leads to psychological distress and psychological distress mediates the relationship between exploitative leadership and knowledge hiding (Guo et al., 2021). Therefore, psychological distress occurs as a mediation in the relationship between exploitative leadership and knowledge hiding. Likewise, exploitative leadership possesses dehumanizing characteristics, such as manipulating and exerting pressure on subordinates, acting egoistically, hindering development, consistently working under challenging conditions, overburdening subordinates, and taking credit for subordinates’ achievements (Schmid et al., 2019; Wu et al., 2021).

Therefore, we investigated subordinates’ knowledge-hiding behavior as an outcome of exploitative leadership through psychological distress. To increase the strength of the influence of exploitative leadership on psychological distress, we examined the moderating effect of leader incivility. We also tested the moderated mediating effect of leader incivility on the mediating effect of psychological distress on the relationship between exploitative leadership and knowledge hiding.

To address these issues, we present the purpose of the current research and its multiple contributions to the literature. First, most previous studies have classified knowledge hiding into a one-factor dimension (Jahanzeb et al., 2019; Moh’d et al., 2021; Islam et al., 2022; Koay and Lim, 2022; Xu and Xue, 2023) or three-factor dimensions, namely, evasive hiding, playing dumb, and rationalized hiding (Akhlaghimofrad and Farmanesh, 2021; Wang et al., 2023; Xu et al., 2023). Farooq and Sultana (2021) suggest that knowledge hiding can be divided into four dimensions: playing dumb, evasive hiding, rationalized hiding, and procrastination. However, no empirical studies have been conducted on these four dimensions. To address these limitations and suggestions, we broaden the existing literature on the dimensions related to knowledge hiding. To the best of our knowledge, this is the first empirical study to focus on these four dimensions of knowledge-hiding behavior.

Second, most studies on knowledge hiding have demonstrated its influence on individual and organizational performance (e.g., Jahanzeb et al., 2019; Singh, 2019; Mubarak et al., 2022). Unlike in previous studies, we provide a research model related to the process underlying knowledge hiding, and then verify it.

Third, exploitative leadership is a newly emerging concept and there is little research on exploitative leadership and its effects on knowledge hiding from employees. This can be seen as a gap in the logical aspect of how exploitative leadership causes knowledge hiding. To fill these gaps, this study aims to explain the relationship via social exchange theory and conservation of resource theory. Furthermore, knowledge hiding generally consists of three subdimensions, However, we consider that other factors may exist. Thus, this study explores another dimension more broadly. This can reduce the gap with previous research.

Finally, the main reasons for selecting small and medium-sized enterprises as more suitable for this research are as follows. The concept of exploitative leadership is a newly emerged leadership style. Most of the exploitative leadership research focused on hospitals (Majeed and Fatima, 2020), hotels (Ye et al., 2022), logistics companies (Lyu et al., 2023), and the education sector (Akhtar et al., 2022). The representative characteristics of exploitative leadership are selfishness, exploiting members to achieve goals, and regarding members as a tool to achieve the leader’s personal goals. In particular, small and medium-sized businesses are characterized by an emphasis on competition and performance orientation. Nevertheless, there is as yet little previous research on exploitative leadership in small and medium-sized enterprises. Therefore, it will be worth revealing the level and influence of exploitative leadership in small and medium-sized businesses.

In addition, most studies have focused on students (Zhai et al., 2020), professors (Demirkasimoglu, 2016), and universities (Yang and Ribiere, 2020) to test knowledge hiding. In addition to educational institutions, leveraging knowledge to maintain or gain a competitive advantage in SMEs is particularly important (Jones and Ratten, 2020; Jafari-Sadeghi et al., 2022). However, research targeting small and medium-sized enterprises is insufficient. In particular, small and medium-sized businesses are demanding higher levels of performance from their employees to ensure competitiveness and sustainability. In this situation, employees’ knowledge plays a fundamental prerequisite for the survival and development of SMEs in the knowledge economy era (Jafari-Sadeghi et al., 2022; Xu et al., 2022). This explains that the importance of knowledge is not only emphasized in educational institutions, but also in small and medium-sized businesses. Nevertheless, employees in SMEs with higher performance anticipations were more likely to be motivated via differential leadership to advance their personal recognition of occupation insecurity and it may then increase employees’ knowledge hiding (Xu et al., 2022). According to these issues, the hiding of knowledge or information related to tasks among SEMs has increased rapidly. Thus, we focus on SME employees to expand the diversity of the sample and identify the main pathways shaping knowledge hiding.

## 2 Theoretical background and hypotheses development

### 2.1 Exploitative leadership and knowledge hiding

Knowledge is one of the most important resources for allowing employees and organizations to achieve success (Pereira and Mohiya, 2021). Hence, organizations encourage their employees to share their knowledge to strengthen organizational competitiveness through creative ideas. However, deliberately hiding knowledge may negatively influence individual and organizational outcomes such as employees’ creativity and organizational innovation.

Most previous studies have divided knowledge hiding into three factors or dimensions (Akhlaghimofrad and Farmanesh, 2021; Wang et al., 2023; Xu et al., 2023). Specifically, the three dimensions of knowledge hiding have been described by Connelly et al. (2012) as: playing dumb (i.e., pretending not to know the requested knowledge of interest), rationalized hiding (i.e., the knowledge owner offers some reasons for not providing knowledge), and evasive hiding (i.e., the knowledge hider provides incomplete or wrong information for knowledge seekers). In addition to these three-factor dimensions of knowledge hiding, Farooq and Sultana (2021) added procrastination for the first time.

The four dimensions of knowledge hiding are summarized as follows. Playing dumbs is defined as pretending not to understand something while requesting specific knowledge or information (Ali et al., 2021). Evasive hiding refers to sharing incorrect information when individuals request it (Hameed et al., 2023). Rationalized hiding is defined as justifying knowledge seekers for failing to offer assistance to a solution. Procrastination refers to a style of behavior that intentionally delays the knowledge requested by a knowledge seeker without explicitly denying it (Farooq and Sultana, 2021).

Knowledge hiding can occur in various ways. In particular, there is playing-dumb behavior in which a person pretends not to understand another person’s requests and conceals knowledge in a situation where knowledge is generally known. Evasive hiding is the act of intentionally conveying incorrect information to avoid conveying knowledge. Furthermore, rationalized hiding is the act that justifies the knowledge seeker’s failure to provide knowledge. In addition to these three types of knowledge-hiding behaviors, another hiding behavior includes the act of deliberately delaying the information of a person requesting knowledge, which is regarded as procrastination. We believe that procrastination, as presented by Farooq and Sultana (2021), is also a type of knowledge hiding. We argue that the conservation of resource theory and social exchange theory may support the underlying linkage between exploitative leadership and knowledge hiding.

Exploitative leadership is the promotion of a leader’s personal self-interest through the exploitation of others (Abdulmuhsin et al., 2021). This includes selfish behavioral traits. In addition, leaders’ selfish behavior can impact employees’ knowledge-hiding behavior (Peng et al., 2019). The reason leadership causes knowledge hiding is to be explained through the conservation of resources theory.

Conservation of resources theory proposes that when individuals are threatened with a potential loss of resources, they respond to such situation by trying to protect their own remaining resources which they possess (Hobfoll, 1989; Byrne et al., 2014; Škerlavaj et al., 2018). The underlying principle of the conservation of resource theory suggests individuals strive to protect, retain, and foster the resources that they value (Hobfoll, 2001; Westman et al., 2004; Akhtar et al., 2022). Employees are susceptible and likely to protect their numerous valued resources ranging from physical resources to energy, and from personal to condition-based (Hobfoll, 1989, 2001).

Furthermore, normally an individual attempts to protect their own valuable resources from further losses when someone encounters threatening situations. Moreover, an exploitative leader can be viewed as a form of workplace stress for subordinates such that they engage in protecting their valued resources (Hobfoll, 1989; Akhtar et al., 2022). In addition, it is highly likely that an exploitative leadership style will ultimately lead employees to perceive incivility. The reason is that awareness of workplace incivility drains individuals motivational mental state for work, after which cognitive effort to preserve their resources and avoid the further loss of personal resource results in subordinates’ knowledge hiding (Escartín et al., 2011; Magee et al., 2017; Obeidat et al., 2018).

In particular, exploitative leaders are a serious menace to their subordinates and take credit for certain of their achievements even if the leader has not contributed to them (Schmid et al., 2019). In addition, the exploitative leader uses personal influence and manipulative behavior against their subordinates for their own benefit. Furthermore, exploitative leadership may evoke a kind of moral justification process that facilitates increased subordinate organizational and interpersonal deviance (Lyu et al., 2023). These statements highlight that if subordinates’ resources such as knowledge or information are threatened by their leader, it will be a strong possibility that subordinates will hide their knowledge to protect their knowledge.

In another aspect, social exchange theory explains how exploitative leadership causes knowledge hiding. Social exchange theory presents that social exchange derives from informal relationships and such exchange creates individual feelings of obligation and trust (Blau, 1964; Peng et al., 2014; Feng et al., 2022). In addition, social exchange theory suggests norms of reciprocity that positive norms of reciprocity promote individuals to return positive treatment they receive according to the exchange of positive treatment. On the contrary, the negative norms of reciprocity promote individuals to return negative treatment according to the exchange of negative treatment (Gouldner, 1960; Cropanzano and Mitchell, 2005; Feng et al., 2022). In particular, social exchange theory emphasizes that individuals are willing to share their own information with coworkers as they may expect something of significant worth in return, and owing to such a process are likely to share knowledge in response (Xiao and Cooke, 2019; Dodokh, 2020). Good relationships promote developing shared trust and respect, which leads employees to utilize constructive exchange for supporting information exchange with one another (Zhao et al., 2016). However, exploitative leaders persistently breach reciprocity norms that usually produce positive supervisor-subordinate exchange. Responding to this negative exchange, subordinates feel obliged to react to such unfair treatment from their leader (Moin et al., 2022). Furthermore, the poor connection is bound to trigger negative correspondence in response to past disagreeable encounters and builds a likelihood of knowledge-hiding behavior (Zhao et al., 2016; Dodokh, 2020).

In addition, social exchange theory posits that when exploited, subordinates exhibit counterproductive knowledge behavior such as knowledge hiding and direct it toward coworkers, those coworkers show similar counterproductive knowledge behavior of hiding their knowledge (Feng et al., 2022). Therefore, exploitative leadership possesses strong negative aspects that can result in knowledge hiding owing to creating a poor level of trust by establishing a low level of exchange relationship with subordinates.

Moreover, sustained exposure to leaders’ exploitation leads organizational members to perceive a form of resource that loses personal autonomy and concerns job control because exploitative leaders give both tedious and boring tasks, place inappropriately high job demands, and exert excessive work pressure on employees (Schmid et al., 2018). According to the reasoning behind the cognitive theory related to stress coping and appraisal, employees’ primary appraisal of the process becomes active when they face exploitative leadership, and this process makes them likely to withhold their own knowledge and increase knowledge hiding (Syed et al., 2019). In addition, if leaders show subordinates that personal interest is more important than mutual interests, subordinates citing this is regarded as institutionalized behavior in the organization and preference their personal interests when asking for knowledge and declining to respond appropriately (Peng et al., 2019; Labafi et al., 2022). Furthermore, exploitative leadership can contribute to an increase in the stress level of subordinates, ultimately leading to increased levels of knowledge hiding (Syed et al., 2019). Therefore, exploited subordinates are reluctant to devote their time and energy to other organizational members’ requests and engage in knowledge hiding (Guo et al., 2021).

*H1*: Exploitative leadership has a positive influence on knowledge hiding.

*H1-1*: Exploitative leadership has a positive influence on playing dumb.

*H1-2*: Exploitative leadership has a positive influence on evasive hiding.

*H1-3*: Exploitative leadership has a positive influence on rationalized hiding.

*H1-4*: Exploitative leadership has a positive influence on procrastination.

### 2.2 The mediating role of psychological distress

When leaders indulge in manipulative and exploitative tactics, such as pressurizing, underchallenging, and overburdening subordinates, those subordinates experience psychological stress (Hobfoll, 1989). Exploitative leadership may create a stressful situation in the workplace that causes subordinates to feel a loss of their potential resources, which may lead to psychological distress and facilitate workplace incivility (Kiyani et al., 2021). Furthermore, exploitation increases organizational members’ psychological strain and undermines their well-being (Livne-Ofer et al., 2019; Schmid et al., 2019; Guo et al., 2021). When subordinates experience and recognize their leaders’ intimidating behavior, this may drain their cognitive resources, increasing their level of psychological distress (Mawritz et al., 2014; Hobfoll et al., 2018). Eventually, exploitative leadership aggravates organizational members’ emotional complexity, which may result in greater psychological distress (Fatima and Majeed, 2023; Karatepe et al., 2023). In summary, exploitative leadership exploits subordinates, causing them to experience workplace stress and jeopardizing their psychological well-being. The negative role of exploitative leadership is expected to eventually cause psychological distress in subordinates.

Thus, we draw on the proposition that psychological distress encourages knowledge hiding. Organizational members engage in knowledge hiding to downplay psychological distress and rationalize the ill-treatment of dehumanization (Robinson et al., 2004). Additionally, they may feel that their organizations treat them as robots and care less for their interests, which may create psychological strain and stress (Robinson et al., 2004; Glicken and Robinson, 2013). Psychological distress is the main cause of organizational dehumanization. In the context of an individual’s knowledge requests, knowledge-hiding behaviors may function as a defensive means to address psychological distress. The high level of psychological distress explains why organizational members possess more aversive psychological reactions, including anxiety and tension, which lead to a lack of physical and emotional resources (Garcia et al., 2017, 2018; Park et al., 2018; Guo et al., 2021). In addition, when facing emotional exhaustion and psychological distress, organizational members consider self-protection behaviors, such as knowledge hiding (Yao et al., 2020; Khelladi et al., 2022).

According to the conservation of resources theory, if people face a loss of resources or experience worry, they will experience psychological strain, facilitate preservation, and try to protect their limited resources (Hobfoll, 1989; Hobfoll et al., 2018; Kiyani et al., 2021). Therefore, exploitative leaders who create a non-constructive atmosphere by abusing organizational members’ efforts may contribute to a destructive atmosphere with psychological distress and anxiety, and lead to subordinates hiding their information and knowledge to confront them (Ghanbari and Majooni, 2022). Specifically, workplace bullying can consume lots of employees’ emotional resources leading to psychological distress, after which individuals will form rational cognition and re-examine whether their efforts are worthwhile. Eventually, they will adopt some self-protection behaviors (such as knowledge hiding) to avoid further loss of resources (Escartin et al., 2011; Magee et al., 2017; Obeidat et al., 2018; Yao et al., 2020). Subordinates tend to develop negative mental states that may create psychological distress when their leaders engage in exploitative behaviors. Working under exploitative leadership is not easy, and subordinates are manipulated, overburdened, and pressured (Kiyani et al., 2021).

In sum, exploitative leadership acts as a work stressor that leads to emotional complexity, which depletes employees’ resources. This loss of resources can cause psychological distress (Fatima and Majeed, 2023). Thus, subordinates find it difficult to gain more resources in adverse psychological distress situations; consequently, they hide their knowledge to gain a competitive edge in their workplaces (Javid et al., 2023). These findings suggest that exploitative leadership causes subordinates to hide their knowledge through psychological distress. Given these arguments, we propose that psychological distress mediates the relationship between exploitative leadership and knowledge hiding.

*H2*: Psychological distress mediates the relationship between exploitative leadership and playing dumb.

*H3*: Psychological distress mediates the relationship between exploitative leadership and evasive hiding.

*H4*: Psychological distress mediates the relationship between exploitative leadership and rationalized hiding.

*H5*: Psychological distress mediates the relationship between exploitative leadership and procrastination.

### 2.3 The moderated mediation role of the leader’s incivility

We predicted that subordinates’ psychological distress would be moderated by the interaction between exploitative leadership and leader incivility. The main cause is that leaders’ incivility violates workplace norms. When a leader behaves rudely toward subordinates, the escalation of negative behavior creates a high level of conflict (Torkelson et al., 2016). In addition, leader incivility triggers retaliatory responses from subordinates and leads to behaviors that limit employees’ efforts and commitment to the organization (Thompson et al., 2018). In particular, in SMEs, the level of leader incivility is relatively high and, consequently, subordinates’ psychological distress increases as they become dissatisfied with all aspects of the job and consider turnover more frequently (Cortina et al., 2001).

In addition, leaders’ incivility may lead to social exclusion and injustice and, in the process, negatively impact subordinates’ psychological and physical well-being, eventually leading to negative psychological distress (Caza and Cortina, 2007). This finding highlights the positive association between leader incivility and psychological distress. Therefore, the more insults and mental bullying the subordinate experiences, the higher their level of psychological distress.

The levels of subordinates’ psychological distress will vary depending on the extent to which they experience leader incivility and exploitative leadership. In particular, a leader’s exploitation negatively impacts the work environment within the organization, gives rise to psychological pain, such as dissatisfaction or depression, and aggravates the leader’s incivility. To achieve their own goals, exploitative leaders pressure employees and choose rude behavior and other methods to intimidate their subordinates, which reduces subordinates’ sense of belonging and leads to a loss of internal resources (Kiyani et al., 2021). In addition, exploitative leaders exploit and oppress subordinates for their own benefit, and pursue their own goals by choosing exploitative behavior to achieve more profit (Ogbonnaya et al., 2016).

Thus, the higher the leader’s incivility, the stronger the effect of exploitative leadership on psychological distress. Furthermore, subordinates consume more resources owing to psychological distress and are more likely to experience higher levels of negative mental well-being, which leads them to adopt knowledge-hiding behaviors to prevent further resource loss (Guo et al., 2021).

Psychological distress was used as a parameter in this study. We hypothesized that the stronger the interaction between exploitative leadership and leaders’ incivility, the higher the psychological distress of subordinates would be. This emphasizes that the mediating effect of psychological distress on the relationship between exploitative leadership and knowledge hiding is positively moderated by leader incivility.

*H6*: Leader incivility positively moderates the relationship between exploitative leadership and psychological distress.

*H7*: Leader incivility moderates the mediating effect of psychological distress on the relationship between exploitative leadership and playing dumb.

*H8*: Leader incivility moderates the mediating effect of psychological distress on the relationship between exploitative leadership and evasive hiding.

*H9*: Leader incivility moderates the mediating effect of psychological distress on the relationship between exploitative leadership and rationalized hiding.

*H10*: Leader incivility moderates the mediating effect of psychological distress on the relationship between exploitative leadership and procrastination.

## 3 Research methodology

### 3.1 Respondents’ demographics and procedure

To verify our research model, we examined full-time employees in China. The data were collected from Chinese SMEs. All participants were instructed to refer to their most recent or current employment experience. From the 300 distributed surveys, 300 responses were received (100% response rate), and 287 data points were used for the empirical analysis. Thirteen data points were judged to lack honesty or integrity and were removed. In addition, we secured the validity of the participants’ work experience at their companies by targeting general employees who had worked in their organizations for more than 6 months.

Descriptive statistics for the characteristics of the sample are as follows. The sample comprised 216 men (75.3%) and 71 women (24.7%). The majority of respondents were between 20 and 29 years of age (53.0%). In contrast, the lowest proportion of respondents were over 50 years (5.2%). In relation to service years, 57 (19.9%) respondents had been working in their companies for less than 1 year, 105 (36.6%) had been working for 1–4 years, 40 (13.9%) had been working for 5–6 years, and 85 (29.6%) had been working there for 7 or more years.

### 3.2 Measurements and main variable

To ensure that the Chinese surveys accurately reflected the original measurement in English, all questionnaires were back-translated into Chinese. Three people confirmed the accuracy of all measurements, and one bilingual author translated all measurements into Chinese. Next, the other two additional translated Chinese versions of the measurements were returned to English to verify the accuracy of the Chinese instruments.

Exploitative leadership was assessed using Schmid’s et al. (2019) method. Sample items include “My leader takes it for granted that my work can be used for his or her personal benefit” and “My leader puts me under pressure to reach his or her personal goals.” Respondents were asked about their degree of exploitative leadership and responses were measured on a 7-point scale, ranging from 1 (strongly disagree) to 7 (strongly agree). The Cronbach’s alpha score was 0.937.

Psychological distress is defined as emotional disturbances that are usually caused by an internal or external stressor and often result in conflict that is unresolved (Amjad et al., 2020). We measured psychological distress using a 10-item instrument developed by Kessler et al. (2002). Sample items include “I feel so nervous that nothing could calm me down” and “I feel restless or fidgety.” Respondents’ psychological distress was measured on a 7-point scale ranging from 1 (strongly disagree) to 7 (strongly agree). Cronbach’s alpha for this study was 0.959.

A leader’s incivility refers to abusive, insulting behavior; repeated, unfair sanctions; and intimidating, offensive, or abuse of power that makes subordinates feel humiliated, upset, vulnerable, or threatened, creating stress and undermining their self-confidence (Vessey et al., 2010). To measure supervisors’ incivility, we asked participants to score honestly based on their experience of the leader’s incivility using a 10-item instrument developed by Cortina et al. (2001). Sample items include “My supervisor paid little attention to my statement or showed little interest in my opinion” and “My supervisor made demeaning or derogatory remarks about me.” We used a Likert 7-point scale ranging from 1 (strongly disagree) to 7 (strongly agree) to measure all items. The Cronbach’s alpha score was 0.951.

Knowledge hiding is defined as an organizational member’s intention to retain or hide his/her knowledge when other members demand knowledge (Yang et al., 2021). Knowledge hiding was measured by dividing it into four sub-dimensions: playing dumb, evasive hiding, rationalized hiding, and procrastination. To achieve this, participants were asked to rate their degree of knowledge hiding (playing dumb, evasive hiding, rationalized hiding, and procrastination) behavior on a 7-point Likert scale ranging from 1 (strongly disagree) to 7 (strongly agree). Participants were asked to truthfully provide their actual experiences.

Connelly et al. (2012) divided knowledge hiding into three dimensions: playing dumb, evasive hiding, and rationalized hiding.

Playing dumb is a behavior that ignites or pretends not to know what the knowledge seeker is asking about (Connelly et al., 2012). The four-item scale for measuring playing dumb was adapted from Akhlaghimofrad and Farmanesh (2021). Sample items include “I pretended that I did not know the information” and “I said that I was not very knowledgeable about the topic.” The Cronbach’s alpha score was 0.937.

Evasive hiding refers to providing knowledge seekers with incomplete knowledge or promising knowledge seekers with an absolute answer in the future with no intention of providing knowledge (Connelly et al., 2012). We used an instrument developed by Akhlaghimofrad and Farmanesh (2021) and a four-item scale to measure evasive hiding. Sample items include “I agreed to help him/her but never really intended to” and “I told him/her that I would help him/her later but stalled as much as possible.” The Cronbach’s alpha score was 0.959.

Rationalized hiding is defined as offering justification to organizational members who are knowledge seekers for failing to provide assistance for solutions to problems or questions (Farooq and Sultana, 2021). Rationalized hiding refers to justifying hiding one’s knowledge by giving reasons (Connelly et al., 2012). The four-item scale for measuring rationalized hiding was adapted from Akhlaghimofrad and Farmanesh (2021). Sample items include “I explained that the information is confidential and only available to people on a particular project” and “I told him/her that my boss would not let anyone share this knowledge.” The Cronbach’s alpha score in this study was 0.884.

**Figure 1:**
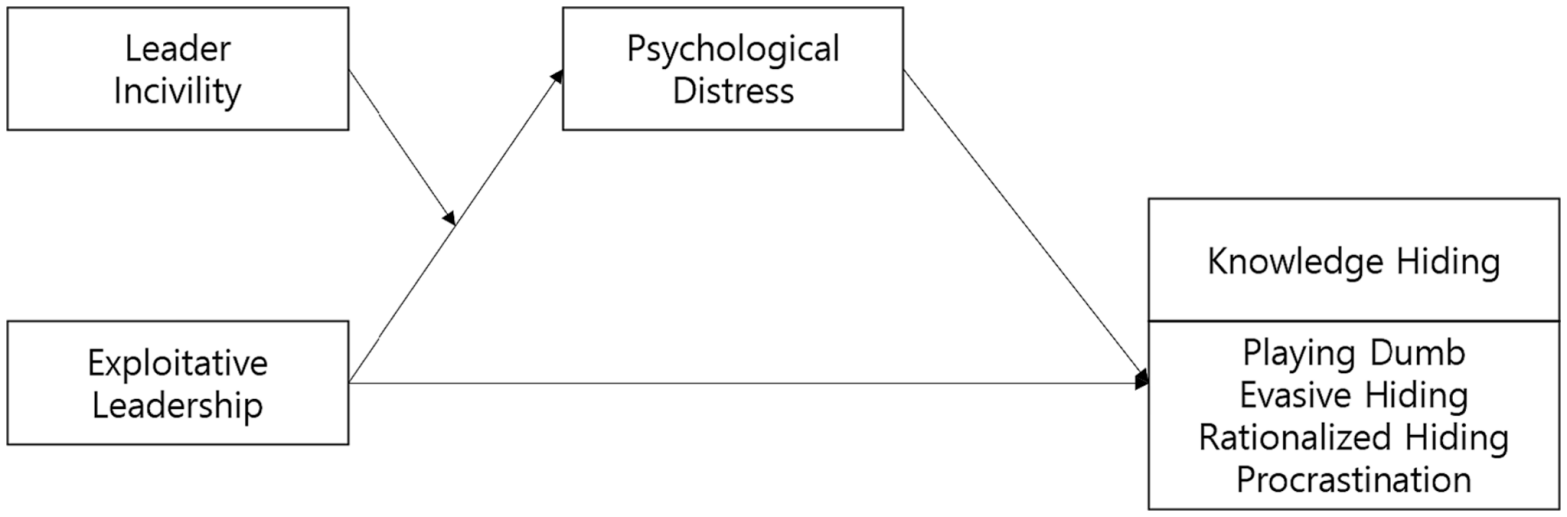
The research model.

Finally, procrastination refers to a style of behavior that intentionally delays the knowledge requested by another member who is a knowledge seeker. It refers to the act of deliberately delaying the knowledge requested by another member without explicitly denying it (Farooq and Sultana, 2021). In order to measure procrastination, of the total 12 items , we used 11 items after thoroughly grasping their overall content. In addition, the content of Steel’s (2010) items was delayed in decision-making. Based on this, the survey was conducted after modifying the content to intentionally delay the time and hide knowledge. Sample items included “I am continually saying ‘I’ll share knowledge with them the next time’” and “In order to avoid sharing knowledge, I often waste time by doing other things.” The Cronbach’s alpha score was 0.955. Figure 1 illustrates the research model.

## 4 Data analysis

### 4.1 Exploratory factor analysis

The results of exploratory factor analysis (EFA) showed that all factor loadings were above 0.50 (with a range of 0.504–0.876). Specifically, psychological distress was loaded as Factor 1, procrastination as Factor 2, leader’s incivility as Factor 3, exploitative leadership as Factor 4, rationalized hiding as Factor 5, evasive hiding as Factor 6, and playing dumb as Factor 7. If the value of the loading factor is above 0.4, it is considered significant (Comrey and Lee, 1992). Furthermore, the result of the Kaiser-Meyer-Olkin (KMO) was 0.945, and Bartlett’s test of sphericity was significant (*p* < 0.001). The results revealed that the factor loadings effectively represented the factors. Table 1 shows the results of EFA.

**Table 1:** Exploratory factor analysis.

Variable	Item	Components						
		1	2	3	4	5	6	7
Exploitative leadership (A)	A1	0.092	0.111	0.135	0.791	0.040	0.065	0.186
	A2	0.097	0.072	0.152	0.876	0.130	0.061	0.004
	A3	0.133	0.114	0.156	0.875	0.065	0.050	0.150
	A4	0.147	0.092	0.206	0.854	0.026	0.145	0.055
	A5	0.211	0.136	0.221	0.853	0.064	0.050	−0.022
Psychological distress (B)	B1	0.797	0.206	0.112	0.052	0.088	0.055	0.224
	B2	0.838	0.217	0.142	0.166	0.067	0.063	0.074
	B3	0.815	0.145	0.235	0.165	0.012	0.185	−0.004
	B4	0.833	0.178	0.158	0.078	0.054	0.011	0.128
	B5	0.853	0.185	0.160	0.057	0.086	0.133	−0.014
	B6	0.759	0.163	0.072	0.097	0.144	−0.019	0.257
	B7	0.787	0.226	0.138	0.117	0.082	0.082	0.127
	B8	0.707	0.227	0.194	0.104	−0.028	0.183	−0.005
	B9	0.784	0.252	0.140	0.082	0.148	0.134	0.057
	B10	0.759	0.210	0.182	0.130	0.150	0.212	0.031
Playing dumb (C)	C1	0.205	0.302	0.206	0.143	0.295	0.144	0.745
	C2	0.151	0.303	0.256	0.176	0.248	0.175	0.746
	C3	0.156	0.278	0.204	0.102	0.238	0.248	0.773
	C4	0.194	0.289	0.224	0.096	0.340	0.159	0.716
Evasive hiding (D)	D1	0.200	0.297	0.262	0.102	0.212	0.691	0.313
	D2	0.230	0.339	0.268	0.139	0.158	0.746	0.216
	D3	0.199	0.363	0.260	0.149	0.173	0.741	0.198
	D4	0.210	0.313	0.283	0.120	0.171	0.780	0.178
Rationalized hiding (E)	E1	0.140	0.122	0.077	0.098	0.840	0.047	0.238
	E2	0.054	0.107	0.064	0.070	0.871	0.038	0.246
	E3	0.077	0.196	0.093	0.052	0.815	0.142	0.204
	E4	0.183	0.191	0.123	0.107	0.614	0.319	0.007
Leader’s incivility (F)	F1	0.322	0.246	0.712	0.321	0.104	0.112	0.276
	F2	0.292	0.248	0.698	0.309	0.108	0.112	0.263
	F3	0.332	0.259	0.731	0.296	−0.008	0.217	0.152
	F4	0.295	0.310	0.728	0.253	0.067	0.290	0.052
	F5	0.247	0.255	0.744	0.235	0.148	0.233	0.087
	F6	0.265	0.303	0.723	0.274	0.041	0.237	0.159
	F7	0.156	0.080	0.604	0.053	0.378	0.128	0.222
Procrastination (G)	G1	0.278	0.735	0.071	0.133	0.052	0.224	0.236
	G2	0.265	0.775	0.073	0.121	0.077	0.194	0.216
	G3	0.285	0.797	0.102	0.125	0.101	0.114	0.238
	G4	0.320	0.789	0.162	0.123	0.054	0.114	0.217
	G5	0.215	0.806	0.172	0.127	0.104	0.212	0.149
	G6	0.189	0.798	0.181	0.095	0.160	0.086	0.154
	G7	0.166	0.830	0.187	0.037	0.060	0.029	0.165
	G8	0.216	0.831	0.192	0.085	0.136	0.077	0.126
	G9	0.240	0.700	0.203	0.114	0.156	0.361	−0.090
	G10	0.189	0.504	0.219	0.072	0.258	0.349	−0.089
	G11	0.103	0.504	0.234	−0.020	0.366	0.196	−0.011
Eigenvalues		7.908	7.858	4.758	4.504	3.575	3.397	3.379
Dispersion (%)		17.573	17.461	10.573	10.009	7.944	7.549	7.510
Cumulative (%)		17.573	35.035	45.608	55.617	63.562	71.110	78.620
KMO = 0.945 (sig = 0.000)								

Factor loadings are above 0.50 (with a range of 0.504–0.876). Factor1: psychological distress; Factor2: Procrastination; Factor3: Leader’s incivility; Factor4: Exploitative leadership; Factor5: Rationalized hiding; Factor6: Evasive hiding; Factor7: Playing dumb. Loading factors on items are shaded dark gray.

### 4.2 Confirmatory factor analysis and validity analysis

A confirmatory factor analysis (CFA) was conducted to determine the goodness of model fit. Five models were tested, and the model fit index was compared.

Model 1 was our research model, in which all variables were input simultaneously and loaded independently. The results revealed *X*^2^ (*p*) = 2359.159(0.000), *X*^2^/df = 2.595, RMSEA = 0.075, IFI = 0.902, CFI = 0.901, PNFI = 0.780, and PGFI = 0.630. that model 1 showed the best model fit compared to the other four alternative models. These results indicate that Model 1 had the best fit index. Table 2 summarizes the results of the structural model.

**Table 2:** Summary of the results of the structural model fit.

Model	*χ*^2^(*p*)	*χ*^2^/df	RMSEA	IFI	CFI	PNFI	PGFI
Model 1 (Expected model of seven-factor^a^)	2359.159 (0.000)	2.595	0.075	0.902	0.901	0.780	0.630
Model 2 (Six-factor^b^)	2890.113 (0.000)	3.292	0.090	0.859	0.859	0.751	0.589
Model 3 (Six-factor^c^)	4100.774 (0.000)	4.438	0.110	0.785	0.784	0.689	0.450
Model 4 (five-factor^d^)	4938.382 (0.000)	5.322	0.123	0.728	0.727	0.642	0.394
Model 5 (four-factor^e^)	4537.496 (0.000)	4.905	0.117	0.755	0.754	0.664	0.468

^a^Exploitative Leadership, Psychological Distress, Supervisor’s Incivility, Playing Dumb, Evasive Hiding, Rationalized Hiding, Procrastination. ^b^Exploitative Leadership and Supervisor’s Incivility, Psychological Distress, Playing Dumb, Evasive Hiding, Rationalized Hiding, Procrastination. ^c^Exploitative Leadership and Psychological Distress, Supervisor’s Incivility, Playing Dumb, Evasive Hiding, Rationalized Hiding, Procrastination. ^d^Playing Dumb and Evasive Hiding and Rationalized Hiding, Exploitative Leadership, Psychological Distress, Supervisor’s Incivility, Procrastination. ^e^Playing Dumb and Evasive Hiding and Rationalized Hiding, Procrastination, Exploitative Leadership, Psychological Distress, Supervisor incivility.

Table 3 presents the results of the CFA and the convergent validity analysis. As shown, the research model showed a good fit for our data with *X*^2^ (*p*) = 2359.159(0.000), *X*^2^/df = 2.595, RMSEA = 0.075, IFI = 0.902, CFI = 0.901, PGFI = 0.630, and PNFI = 0.780. These indices satisfy the acceptable requirement (Jin and Hahm, 2021; Jin et al., 2022). Moreover, the standardized regression weights of exploitative leadership, psychological distress, leader’s incivility, playing dumb, evasive hiding, rationalized hiding, and procrastination were all higher than 0.5. These results indicated that all items loaded significantly on the factors.

**Table 3:** The results of confirmatory factor analysis and convergent validity analysis.

Variables		Estimate	SE	CR	*p*	Standardized regression weights	AVE	C.R
Exploitative leadership(A)	A1	1				0.645	0.600	0.722
	A2	1.489	0.084	17.716	***	0.831		
	A3	1.463	0.077	18.913	***	0.863		
	A4	1.298	0.078	16.713	***	0.803		
	A5	1.307	0.094	13.854	***	0.711		
Psychological distress(B)	B1	1				0.710	0.624	0.884
	B2	1.045	0.053	19.667	***	0.760		
	B3	1.099	0.078	14.13	***	0.700		
	B4	1.166	0.068	17.27	***	0.798		
	B5	1.120	0.072	15.506	***	0.747		
	B6	1.256	0.066	18.908	***	0.835		
	B7	1.217	0.066	18.458	***	0.823		
	B8	1.305	0.068	19.335	***	0.847		
	B9	1.263	0.063	20.169	***	0.861		
	B10	1.153	0.065	17.665	***	0.803		
Playing dumb (C)	C1	1				0.754	0.745	0.859
	C2	1.236	0.056	21.91	***	0.89		
	C3	1.297	0.057	22.71	***	0.905		
	C4	1.295	0.058	22.175	***	0.895		
Evasive hiding(D)	D1	1				0.770	0.721	0.854
	D2	1.15	0.046	24.915	***	0.887		
	D3	1.145	0.044	25.774	***	0.864		
	D4	1.221	0.059	20.603	***	0.871		
Rationalized hiding(E)	E1	1				0.629	0.667	0.763
	E2	1.282	0.073	17.593	***	0.825		
	E3	1.407	0.069	20.401	***	0.928		
	E4	1.307	0.07	18.594	***	0.854		
Leader’s incivility (F)	F1	1				0.551	0.694	0.852
	F2	1.335	0.066	20.151	***	0.876		
	F3	1.315	0.072	18.171	***	0.839		
	F4	1.37	0.066	20.897	***	0.896		
	F5	1.395	0.065	21.388	***	0.926		
	F6	1.307	0.072	18.244	***	0.831		
	F7	1.376	0.072	19.1	***	0.855		
Procrastination(G)	G1	1				0.561	0.628	0.878
	G2	0.94	0.076	12.393	***	0.580		
	G3	1.153	0.072	16.056	***	0.742		
	G4	1.298	0.069	18.937	***	0.848		
	G5	1.264	0.074	17.174	***	0.802		
	G6	1.335	0.072	18.621	***	0.843		
	G7	1.329	0.066	20.122	***	0.876		
	G8	1.315	0.066	19.869	***	0.871		
	G9	1.309	0.068	19.376	***	0.860		
	G10	1.28	0.067	19.043	***	0.854		
	G11	1.232	0.072	17.053	***	0.801		
Model fit index	*X*^2^ (*p*) = 2359.159(0.000), X2/df = 2.595, RMSEA = 0.075, IFI = 0.902, CFI = 0.901, PGFI = 0.630, PNFI = 0.780							

Exploitative leadership (AVE = 0.600, C.R = 0.722), Psychological distress (AVE = 0.624, C.R = 0.884), Playing dumb (AVE = 0.745, C.R = 0.859), Evasive hiding (AVE = 0.721, C.R = 0.854), Rationalized hiding (AVE = 0.667, C.R = 0.763), Leader’s incivility (AVE = 0.694, C.R = 0.852), Procrastination (AVE = 0.628, C.R = 0.878).

In addition, we calculated the average variance extracted (AVE) and composite reliability (CR) to verify convergent validity. The AVE for exploitative leadership, psychological distress, leader incivility, playing dumb, evasive hiding, rationalized hiding, and procrastination were all greater than 0.6. CR for exploitative leadership, psychological distress, leader’s incivility, playing dumb, evasive hiding, rationalized hiding, and procrastination was all higher than 0.7. Overall, the value of AVE and CR satisfied acceptable requirements (Jin and Hahm, 2021; Jin et al., 2022). Thus, all measurements had significant validity.

After testing and checking convergent validity, we used the Fornell-Larcker criterion to investigate the significance of discriminant validity. The results showed that the AVE value was greater than the square of the correlation for each set of constructs. Furthermore, the square root of the AVE for a given construct was higher than the absolute value of the squared correlation of the construct with other factors. Specifically, all square root values of the AVE for all constructs were higher than the correlation between the construct and the other constructs in the current model. According to these results, discriminant validity was significant and supported. Table 4 shows the results of discriminant validity.

**Table 4:** The results of discriminant validity.

	1	2	3	4	5	6	7
1	**(0.775)**						
2	0.121	**(0.790)**					
3	0.298	0.346	**(0.833)**				
4	0.121	0.194	0.362	**(0.863)**			
5	0.128	0.243	0.454	0.370	**(0.849)**		
6	0.057	0.100	0.166	0.320	0.232	**(0.817)**	
7	0.110	0.316	0.372	0.355	0.445	0.186	**(0.792)**

1 = Exploitative Leadership, 2 = Psychological Distress, 3 = Supervisor’s incivility, 4 = Playing Dumb, 5 = Evasive Hiding, 6 = Rationalized Hiding, 7 = Procrastination. Each diagonal element (bolded) is the AVE value, and the other elements are the correlation squares between variables.

### 4.3 Test of common method variance

Common method variance (CMV) is an issue that could threaten the validity of linkage outcomes between constructs (Williams and Brown, 1994; Reio, 2010).

In order to test for CMV, we performed EFA and calculated both the eigenvalues and total variance. Psychological distress was factor 1 and accounted for 17.573%, procrastination was factor 2 and accounted for 17.461%, leaders’ incivility was factor 3 and accounted for 10.573, exploitative leadership was factor 4 and accounted for 10.009%, rationalized hiding was factor 5 and accounted for 7.944%, evasive hiding was factor 6 and accounted for 7.549%, and playing dumb was factor 7 and accounted for 7.510% of the variance. All eigenvalues were greater than 1 and the value of the total variance was lower than 40%. These results indicate that CMV was not a problem in this study.

### 4.4 Test of reliability

To validate the measures, we evaluated Cronbach’s alpha for each latent construct. As shown in Table 4, all Cronbach’s alpha values were acceptable and each value was higher than 0.70, as suggested by Nunnally (1978). Specifically, Cronbach’s alpha values were as follows: exploitative leadership, 0.937; psychological distress, 0.959; supervisor’s incivility, 0.951; playing dumb, 0.956; evasive hiding, 0.959; rationalized hiding, 0.884; and procrastination, 0.955. As presented in Table 5, Cronbach’s alpha values for all the research variables were higher than the acceptable level of 0.70.

**Table 5:** Results of the reliability analysis.

Variable	Items	Cronbach’s alpha
Exploitative leadership	1. My leader takes it for granted that my work can be used for his or her personal benefit.	0.937
	2. My leader benefits from my work without sharing the praise.	
	3. My leader puts me under pressure to reach his or her personal goals.	
	4. My leader plays my colleagues and me off against each other to reach his or her goals.	
	5. My leader does not allow me to develop professionally, as his or her goals are the priority.	
Psychological distress	1. I feel depressed.	0.959
	2. I feel so depressed that nothing could cheer me up.	
	3. I feel hopeless.	
	4. I feel restless or fidgety.	
	5. I feel so restless that I could not sit still.	
	6. I feel tired out for no good reason.	
	7. I feel that everything is an effort.	
	8. I feel worthless.	
	9. I feel nervous.	
	10. I feel so nervous that nothing could calm me down.	
Leader’s incivility	1. My leader puts me down or is condescending to me.	0.951
	2. My leader pays little attention to my statement or shows little interest in my opinion.	
	3. My leader makes demeaning or derogatory remarks about me.	
	My leader addresses me in unprofessional terms, either publicly or privately.	
	5. My leader ignores or excludes me from professional camaraderie.	
	6. My leader doubts my judgment on a matter over which I have responsibility.	
	7. My leader makes unwanted attempts to draw me into a discussion of personal matters.	
Playing dumb	1. I pretended that I did not have the knowledge.	0.956
	2. I said that I did not know, even though I did.	
	3. I pretended I did not know what s/he was talking about.	
	4. I said that I was not very knowledgeable about the topic.	
Evasive hiding	1. I agreed to help him/her but never really intended to.	0.959
	2. I agreed to help him/her but instead gave him/her different information from what she/he wanted.	
	3. I told him/her that I would help him/her out later but stalled as much as possible.	
	4. I offered him/her some other information instead of what he/she really wanted.	
Rationalized hiding	1. I explained that I would like to tell him/her, but was not supposed to.	0.884
	2. I explained that the information is confidential and only available to people on a particular project.	
	3. I told him/her that my boss would not let anyone share this knowledge.	
	4. I said that I would not answer his/her questions.	
Procrastination	1. I delay sharing knowledge related to my task until it’s too late.	0.955
	2. Even after I share the knowledge, I delay acting upon it.	
	3. I waste a lot of time on trivial matters before sharing the knowledge.	
	4. In preparation for some deadlines, I often waste time by doing other things.	
	5. Rarely have I been able to convey knowledge to others within a few days.	
	6. I often find myself sharing knowledge with people that I had intended to do days before.	
	7. I am continually saying “I’ll share knowledge the next time.”	
	8. I generally delay before sharing the knowledge I have to.	
	9. I find myself running out of time.	
	10. I do not tell knowledge done on time.	
	11. I do not tell knowledge deadlines.	

Exploitative leadership = 0.937, Psychological distress = 0.959, Leader’s incivility = 0.951, Playing dumb = 0.956, Evasive hiding = 0.959, Rationalized hiding = 0.885, Procrastination = 0.955.

### 4.5 Descriptive statistics and correlation analysis

Descriptive statistics and Pearson’s correlation analyzes were performed using SPSS 26.0 and the results are shown in Table 6. The results of the correlation analysis showed that exploitative leadership was positively related to psychological distress, leader incivility, playing dumb, evasive hiding, rationalized hiding, and procrastination. Additionally, psychological distress was positively related to leaders’ incivility, playing dumb, evasive hiding, rationalized hiding, and procrastination. Finally, leaders’ incivility was positively related to playing dumb, evasive hiding, rationalized hiding, and procrastination. Therefore, all correlations between variables were statistically significant.

**Table 6:** The results of descriptive statistics and correlation between variables.

	Mean	SD	1	2	3	4	5	6	7
1	3.946	1.840	–						
2	3.865	1.409	0.349***	–					
3	3.254	1.575	0.546***	0.588***	–				
4	4.036	1.629	0.348***	0.441***	0.602***	–			
5	3.213	1.559	0.358***	0.493***	0.674***	0.608***	–		
6	4.132	1.402	0.238***	0.316***	0.407***	0.566***	0.482***	–	
7	3.614	1.389	0.332***	0.562***	0.610***	0.596***	0.667***	0.431***	-

1 = Exploitative Leadership 2 = Psychological Distress 3 = Leader’s Incivility 4 = Dumb Playing 5 = Evasive Hiding 6 = Rationalized Hiding 7 = Procrastination. ***p < 0.001, **p < 0.01, *p < 0.05.

### 4.6 Hypotheses testing

A total of six hypotheses were formulated in this study. We used SPSS PROCESS Macro version 3.4, specifically, Models 3 and 7, to test the hypotheses. The summary of the hypothesis testing results is as follows. Exploitative leadership positively influenced playing dumb (estimate = 0.196, *p* < 0.001, Boot LLCI = 0.100, Boot ULCI = 0.293), evasive hiding (estimate = 0.180, p < 0.001, Boot LLCI = 0.090, Boot ULCI = 0.269), rationalized hiding (estimate = 0.111, p < 0.001, Boot LLCI = 0.022, Boot ULCI = 0.200), and procrastination (estimate = 0.117, p < 0.001, Boot LLCI = 0.080, Boot ULCI = 0.205). This finding indicated that H1, H1-1, H1-2, H1-3, and H1-4 were supported. In relation to testing the mediating effect of psychological distress, we performed a bootstrapping analysis and used a sample of 5,000. We utilized a 95% CI for the indirect effect of psychological distress.

The results showed that the mediating effect of psychological distress on the relationship between exploitative leadership and playing dumb was 0.112 with Boot 95% CI (0.060, 0.187), evasive hiding was 0.124 with Boot 95% CI(0.073, 0.195), rationalized hiding was 0.071 with Boot 95% CI (0.025, 0.130), and procrastination was 0.134 with Boot 95% CI (0.080, 0.205). Therefore, H2, H3, H4, and H5 were supported. Table 7 shows the results of the hypotheses testing for the structured model.

**Table 7:** Hypotheses testing for the structured model.

Path			Estimate	Boot 95% CI	*p*
Exploitative leadership	→	Psychological Distress	0.267	(0.183, 0.351)	***
Psychological distress	→	Playing Dumb	0.420	(0.294, 0.546)	***
Exploitative leadership	→	Playing Dumb	0.196	(0.100, 0.293)	***
Psychological distress	→	Evasive Hiding	0.464	(0.348, 0.581)	***
Exploitative leadership	→	Evasive Hiding	0.180	(0.090, 0.269)	***
Psychological distress	→	Rationalized Hiding	0.264	(0.148, 0.380)	***
Exploitative leadership	→	Rationalized Hiding	0.111	(0.022, 0.200)	***
Psychological distress	→	Procrastination	0.501	(0.401, 0.601)	***
Exploitative leadership	→	Procrastination	0.117	(0.080, 0.205)	***

Mediating effect	Indirect effect	Boot 95% CI	Significant
Exploitative Leadership → Psychological Distress → Playing Dumb	0.112	(0.060, 0.187)	***
Exploitative Leadership → Psychological Distress → Evasive Hiding	0.124	(0.073, 0.195)	***
Exploitative Leadership → Psychological Distress → Rationalized Hiding	0.071	(0.025, 0.130)	***
Exploitative Leadership → Psychological Distress → Procrastination	0.134	(0.080, 0.205)	***

Hypotheses were tested by using path analysis.

According to Hair and Sarstedt (2019), partial least squares structural equation modeling (PLS-SEM) is more appropriate than structural equation modeling (SEM) for demonstrating multiple relationships. PLS-SEM was conducted to verify the significance of the mediating effect of psychological distress with both the indirect effect and variance accounted for (VAF) and the results are shown in Table 8. The results showed the indirect effect of psychological distress on the relationship between exploitative leadership and playing dumb (indirect effect = 0.112, total effect = 0.308, and VAF = 36.4%), evasive hiding (indirect effect = 0.124, total effect = 0.304, and VAF = 40.8%), rationalized hiding (indirect effect = 0.070, total effect = 0.181, and VAF = 38.7%), and procrastination (indirect effect = 0.134, total effect = 0.251, and VAF = 53.4%). A VAF below 20% signifies insignificant mediation, a VAF from 20 to 80% signifies partial mediation, and a VAF above 80% is regarded as full mediation, as suggested by Hair et al. (2014). In the current study, VAF was in the range of 20–80%. Thus, psychological distress showed partial mediation, and H6, H7, H8, and H9 were significantly supported.

**Table 8:** Mediating analysis in PLS-SEM.

Effect	Path	Path coefficient	Indirect effect	Total effect	VAF	*p*
Indirect with mediator	EL → PD	0.196	–	0.308	36.4%	***
	EL → PDS	0.267	0.112			
	PDS → PD	0.420				
Indirect with mediator	EL → EH	0.180	–	0.304	40.8%	***
	EL → PDS	0.267	0.124			
	PDS → EH	0.464				
Indirect with mediator	EL → RH	0.111	–	0.181	38.7%	***
	EL → PDS	0.267	0.070			
	PDS → RH	0.264				
Indirect with mediator	EL → PC	0.117	–	0.251	53.4%	***
	EL → PDS	0.267	0.134			
	PDS → PC	0.501				

EL = Exploitative Leadership, PDS=Psychological Distress，PD=Playing Dumb, EH = Evasive Hiding, RH = Rationalized Hiding, PC=Procrastination. ***p < 0.001, **p < 0.01, *p < 0.05.

In order to test the moderating effect of leaders’ incivility, we performed a bootstrapping analysis using a sample size of 5,000 and a 95% CI. The moderating effect of leaders’ incivility was statistically significant (estimate = 0.052, *p* < 0.05). Moreover, the CI for the moderating effect of leaders’ incivility on the relationship between exploitative leadership and psychological distress did not include zero (95% CI: [0.011–0.094]). Therefore, the moderating effect of leaders’ incivility was statistically significant, and H6 was supported. Table 9 shows the moderating effect of leader incivility.

**Table 9:** The moderating effect of leader’s incivility.

Variable	Estimate	SE	t	*p*	Boot LLCI	Boot ULCI
Exploitative leadership (A)	−0.131	0.078	−1.676	0.095	−0.286	0.023
Leader’s incivility (B)	0.282	0.104	2.710	0.007	0.077	0.487
Interaction (A x b)	0.052	0.021	2.472	0.014	0.011	0.094

The moderating effect of psychological distress.

**Figure 2:**
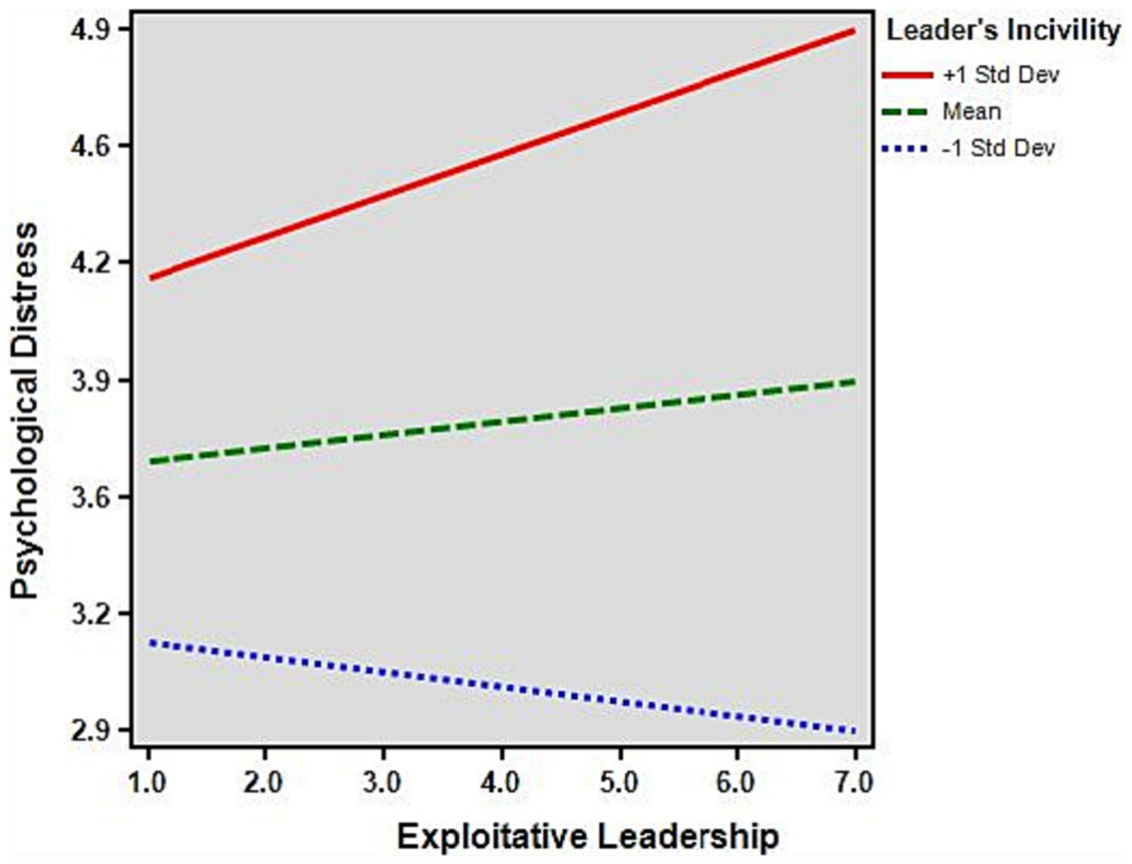
The moderating effect of psychological distress. Leader incivility positively moderates the relationship between exploitative leadership and psychological distress.

Figure 2 illustrates the moderating effect of leader incivility. The graph shows that exploitative leaders reported higher levels of psychological distress when subordinates’ recognition of their leaders’ incivility increased. By contrast, exploitative leaders reported lower levels of psychological distress when subordinates’ recognition of leaders’ incivility was lower.

The moderated mediation model was examined using SPSS PROCESS Macro 3.4 version with Model 7 and tested using 95% CI and 5,000 bootstrapping re-samples.

The results showed that moderated mediation effect of leaders’ incivility on the mediating effect of psychological distress on the relationship between exploitative leadership and playing dumb [index = 0.022, 95% CI = (−0.002, 0.050)], evasive hiding [index = 0.024, 95% CI = (−0.004, 0.055)], rationalized hiding [index = 0.014, 95% CI = (−0.001, 0.037)], and procrastination [index = 0.026, 95% CI = (−0.004, 0.057)]. These findings indicate that H7, H8, H9, and H10 were rejected. Table 10 presents the moderated mediating effect of leader incivility.

**Table 10:** The moderated mediation effect of leader’s incivility.

Index of moderated mediation				
Path (Independent Variable → Mediator → Dependent Variable)	Moderator	Index	Boot SE	Boot 95% CI
Exploitative Leadership → Psychological Distress → Playing Dumb	Leader’s incivility	0.022	0.014	(−0.002, 0.050)
Exploitative Leadership → Psychological Distress → Evasive Hiding	Leader’s incivility	0.024	0.015	(−0.004, 0.055)
Exploitative Leadership → Psychological Distress → Rationalized Hiding	Leader’s incivility	0.014	0.010	(−0.001, 0.037)
Exploitative Leadership → Psychological Distress → Procrastination	Leader’s incivility	0.026	0.015	(−0.004, 0.057)

The moderated mediation effect of leader incivility on the relationship between exploitative leadership and playing dumb, evasive hiding, rationalized hiding, and procrastination.

## 5 Discussion

This study examined how exploitative leadership influences knowledge-hiding behavior. Specifically, we verified the mediating effect of psychological distress on the relationship between exploitative leadership and knowledge hiding. Furthermore, the moderating effect of leader incivility in the relationship between exploitative leadership and psychological distress was tested. This study contributed to expanding the research scope on exploitative leadership and knowledge hiding. Furthermore, the impact of exploitative leadership on knowledge-hiding behavior was presented through social exchange theory and conservation of resource theory. Existing research is insufficient to verify the causal relationship between exploitative leadership and knowledge hiding. Therefore, this study contributes to filling the gap.

This study is the first to empirically test the four dimensions (playing dumb, evasive hiding, rationalized hiding, and procrastination) of knowledge hiding, contributing to expanding the research dimensions of knowledge-hiding behavior by additionally verifying the subdimensions of knowledge hiding. Furthermore, unlike most previous studies that explored or verified only the antecedents of knowledge hiding and demonstrated only mediating or moderating effects, we verified the process leading to knowledge hiding and the significance of the research model. Finally, while most previous studies have statistically tested knowledge hiding in educational institutions, we emphasized the negative aspects of knowledge hiding among Chinese SMEs’ employees, contributing to a deeper understanding of the causes and processes that lead to knowledge hiding in diverse industries.

## 6 Conclusion and implications

### 6.1 Conclusion

To extend the understanding of how exploitative leadership leads to knowledge hiding, we emphasized and explained the role of subordinates’ psychological distress and verified its impact on the relationship between exploitative leadership and knowledge hiding. This study provides new insights into knowledge-hiding behaviors. The main purpose of our research was to explore and determine whether exploitative leadership is the main factor in creating subordinates’ knowledge hiding in Chinese SEMs. Further, we aimed to expand the dimensions of knowledge hiding. Most previous studies have focused on the three dimensions (playing dumb, evasive hiding, and rationalized hiding) of knowledge hiding, and we added procrastination as presented by Farooq and Sultana (2021).

We revealed a mechanism that transmits the influence of exploitative leadership on knowledge hiding. Exploitative leadership positively influenced knowledge hiding. In addition, psychological distress mediated the relationship between exploitative leadership and knowledge hiding. Furthermore, leader incivility positively moderated the relationship between exploitative leadership and psychological distress. Moreover, we expected leaders’ incivility to positively moderate the mediating effect of psychological distress on the relationship between exploitative leadership and knowledge hiding. However, the moderated mediation effect of leader incivility was insignificant.

### 6.2 Theoretical implications

We include several contributions to theory. First, this study explained how exploitative leadership increases knowledge hiding through conservation of resource theory and social exchange theory.

We contribute to the literature on exploitative leadership by identifying its outcome as knowledge hiding. Exploitative leadership is a new concept, that most previous research on focused on the relationship to turnover intention (Iqbal et al., 2022), employees’ proactive customer service performance (Sun et al., 2023), organizational cynicism (Elsaied, 2022), and innovative behavior (Wang et al., 2021, 2023a) as a reaction to exploitative leadership. However, there is little research examining the human relationship between exploitative leadership and knowledge hiding. Therefore, we verified the outcome of another aspect of exploitative leadership and presented the theories on how it leads to knowledge hiding. In relation to the casual relationship between exploitative leadership and knowledge hiding, we provide conservation of resource theory and social exchange theory. The underlying principle of the conservation of resource theory suggests individuals strive to protect, retain, and foster the resources that they value (Hobfoll, 2001; Westman et al., 2004; Akhtar et al., 2022). Therefore, exploitative leadership makes members feel greatly threatened, which ultimately leads them to hide knowledge. In addition, social exchange theory presents norms of reciprocity, and negative norms of reciprocity promote individuals to return negative treatment in exchange for negative treatment (Gouldner, 1960; Cropanzano and Mitchell, 2005; Feng et al., 2022). Therefore, the poor interpersonal connections are bound to trigger negative correspondence as a reaction to past disagreeable encounters and create knowledge hiding (Zhao et al., 2016; Dodokh, 2020). According to this, we consider to have filled an important gap in the literature around exploitative leadership and knowledge hiding.

Second, there is little research on how exploitative leadership causes knowledge hiding. In addition, it can be seen that it is important to reveal the process that exploitative leadership causes knowledge hiding and to derive logical aspects. This is regarded as a theoretical gap in the process of how exploitative leadership leads to knowledge hiding. To fill theoretical gaps, this study logically identified the mediating role of psychological distress and verified its influence. In most previous studies, psychological distress had significant mediating role to work engagement (Anasori et al., 2021), turnover intention (Anasori et al., 2021), safety compliance and safety participation (Mirza et al., 2022), and employee creativity (Raza and Shah, 2023). In particular, research on the mediating effect that causes knowledge hiding via psychological distress is quite insufficient. Therefore, what is meaningful to this research is that it revealed the mediating effect of psychological distress on the relationship between exploitative leadership and knowledge hiding. Specifically, exploitative leadership aggravates individuals’ emotional complexity, resulting in high levels of psychological distress (Fatima and Majeed, 2023; Karatepe et al., 2023). The main reason for this is that if individuals recognize and experience a leader’s intimidating behavior, it will drain their cognitive resources, causing a high level of psychological distress via loss of resources (Mawritz et al., 2014; Hobfoll et al., 2018; Fatima and Majeed, 2023). Thus, exploitative leadership positively influences psychological distress. These results are consistent with those of Guo et al. (2021). Psychological distress is induced by a dehumanizing culture, which explains how organizational members try to abstain from further personal resource depletion and psychological distress, eventually leading to knowledge hiding and defensive behavior (Robinson et al., 2004).

Third, this study demonstrated the mediating effect of psychological distress. However, rather than simply focusing on the mediating effect, additional logic appears to be needed as to what variable moderates the level of psychological distress. Therefore, this study logically identified the moderating roles of workplace incivility and revealed how it moderates psychological distress through exploitative leadership interaction with workplace incivility. The interaction between exploitative leadership and leaders’ incivility revealed differences in the level of psychological distress. The results showed that leader incivility had a positive moderating effect on the relationship between exploitative leadership and psychological distress. Leader incivility creates a stressful workplace climate, causing anxiety and insecurity and leading to low levels of psychological safety (Liu et al., 2016, 2020). Leader incivility reduces individuals’ psychological safety and eventually increases their psychological distress. In addition, the characteristics of exploitative leaders include selfishness, exploitation, discouragement, increased workload, creation of hurdles, and pressurization (Schmid et al., 2019; Kiyani et al., 2021). All these characteristics are expected to have a strong relationship with psychological distress. Leaders’ incivility facilitates social exclusion and injustice and negatively impacts mental and physical health. This may eventually create negative psychological effects such as psychological distress (Caza and Cortina, 2007). This finding suggests that leader incivility and exploitative leadership are positively related. Therefore, subordinates experience a high level of exploitative leadership, which leads to higher levels of leader incivility. In addition, the stronger the leader’s incivility, the greater the subordinates’ perceptions of exploitative leadership. Therefore, the interaction between exploitative leadership and leaders’ incivility leads to higher levels of psychological distress.

Finally, this research is an attempt to bridge exploitative leadership and knowledge hiding and is the first empirical study to expand the dimensions of knowledge-hiding behavior of playing dumb, evasive hiding, rationalized hiding, and procrastination. Knowledge hiding is a type of individual behavior in which individuals intentionally hide useful knowledge or fail to provide substantive knowledge or information to colleagues when requested to do so (Serenko and Bontis, 2016; Chaker et al., 2021). This study divided knowledge hiding into four dimensions (playing dumb, evasive hiding, rationalized hiding, and procrastination) and verified how they interact. The results proved that these four dimensions exist independently. Individuals react to various actions in order to hide their knowledge. In particular, the behavior of deliberately providing incorrect information about knowledge, providing justifications to knowledge seekers, pretending not to understand the knowledge, and deliberately delaying and not revealing the knowledge. In addition to playing dumb, evasive hiding, and rationalized hiding, we suggest that procrastination is a type of knowledge hiding.

### 6.3 Managerial implications

From a practical perspective, the most salient result of our research is the main role of exploitative leadership in shaping perceptions of knowledge hiding and we present managerial implications for SEMs and leaders.

First, as individual resources become more scarce organizations and the value becomes more useful.

This phenomenon can strengthen members’ sense of protection for their own resources and lead to knowledge hiding. We implicate that knowledge hiding is influenced by exploitative leadership.

Since small and medium-sized enterprises value knowledge utilization, knowledge-hiding behavior is a main negative variable that ultimately causes organizational sustainability and development. Therefore, the role and behavior of leaders in an organization must be periodically monitored. In particular, leaders and members should be officially informed of the dangers of exploitative behavior. In addition, exploitative leadership is positively related to organizational commitment, suggesting that subordinates recognize their leader as exploitative and are more likely to create a cynical attitude toward their organizations (Elsaied, 2022). Therefore, organizations should conduct education on the negative consequences that can occur due to exploitation will be needed. The important expectation is to create a culture that emphasizes organizational ethics and the leader’s ethical behavior. Furthermore, organizations should closely monitor the quality of leader - member exchange.

Second, because leader’s roles are important to individual attitude and organizational performance. It indicates that organizations should pay more attention to investigating the leadership style of candidates when organizations select leaders (Schmid et al., 2019). Leaders possess power, and thus the possibility of abusing that power. Hence, it is worth determining whether a leader focuses only on excessive self-interest. Furthermore, candidates who are excessively selfish or only care about their own interests are undesirable leaders and should be evaluated carefully (Judge and LePine, 2007).

Third, Organizations should establish and provide training systems in order to convey both appropriate values and guidelines to leaders. Moreover, Organizations should build proactive feedback systems for leadership and also identify exploitative leaders by using 360-degree detection (Lyu et al., 2023). This can be considered as an important measure to prevent exploitative behavior. By implementing such a system, education, and training, negative problems such as leader incivility, abusive supervision, and exploitative behavior within the organization can be reduced.

Fourth, we emphasize that a high level of knowledge hiding may decrease team performance, innovative behavior, and organizational innovation. Conversely, although knowledge-sharing behaviors among organizational members add value to organizations, they become reluctant, unwilling, and even dispassionate about sharing their knowledge with coworkers (Connelly et al., 2012; Connelly and Zweig, 2015; Ghani et al., 2020). We predict that voluntary knowledge sharing will be beneficial to organizations and that compulsory knowledge sharing may lead to negative outcomes. Therefore, organizations should make employees aware of the importance of teamwork. Teamwork is in groups with a stronger culture of collectivism than individualism. In particular, Chinese organizational culture has a strong tendency to prefer collectivism. Therefore, it is important for leaders and organizations in small and medium-sized businesses to support their employee’s growth. In addition, leaders should share their knowledge with subordinates. It is likely to form an organizational sharing culture over time. This allows employees to freely share their knowledge, thereby reducing knowledge-hiding behavior.

Finally, our findings suggest that unethical leadership is positively associated with psychological distress, which subsequently positively influences knowledge hiding (Qin et al., 2021). Specifically, a high level of job complexity is required to mitigate the influence of unethical leadership on psychological distress, which, in turn, decreases knowledge hiding (Qin et al., 2021). In addition, exploitative leadership is unethical to subordinates because such leaders are solely self-centered and exploit subordinates’ limited resources such as energy and time (Wang et al., 2023b). Overall, psychological distress is a core element of knowledge hiding. The key factor that can lead to knowledge hiding is psychological distress. Psychological distress is particularly closely related to working conditions and working environment. In particular, employees may experience psychological distress depending on the leader’s management style, behavior, and roles. Additionally, overtime working hours and excessive workload are both key variables that can increase employees’ psychological distress. In order to reduce employees’ psychological distress, organizations should pay more attention to employees’ psychological well-being. It will be necessary to check employees’ needs and opinions. In addition, it is important to identify employees’ dissatisfaction and satisfaction elements and manage them in an appropriate manner. Furthermore, organizations should provide opportunities for employees to recognize their value within the organization and to induce internal motivation by providing their work with meaning. This management style is expected to reduce psychological distress and ultimately contribute to organizational performance.

### 6.4 Limitations and future research

Despite these implications, this study has several limitations and directions for future research.

First, the elements that lead to knowledge hiding can be explored from various perspectives. However, this study focuses only on exploitative leadership. We considered two main causes of knowledge hiding involving both interpersonal and organizational aspects. Regarding interpersonal aspects, knowledge-hiding behavior is a strategy among talented organizational members in international SMEs used to continually maintain their own self-efficacy and/or maximize their own control of strategic knowledge (Xiong et al., 2019; Jafari-Sadeghi et al., 2022). Additionally, it is worth considering organizational factors. We argue that leadership, organizational climate, and organizational culture induce knowledge hiding. Therefore, future research should verify the relationship between the aforementioned elements and knowledge hiding.

Second, emerging studies suggest that knowledge-hiding behavior is not simply the opposite of knowledge sharing, and it is crucial to explore both the progress of research and the development trends of knowledge hiding (Xia et al., 2022). Therefore, this study verified the effect of exploitative leadership on knowledge hiding via psychological distress. We only focused on the mediating effects of psychological distress. In this regard, we recommend that future research explore the serial multiple mediation model that causes knowledge hiding and perform more in-depth research.

Third, leader incivility moderates the mediating effect of psychological distress on the relationship between exploitative leadership and knowledge hiding. However, the moderated mediation effect of leader incivility was not significant. Correlation analysis showed that leader incivility was positively related to exploitative leadership, psychological distress, playing dumb, evasive hiding, rationalized hiding, and procrastination. In addition, the moderating effect of leader incivility on the relationship between exploitative leadership and psychological distress was statistically significant. Based on these results, we suggest verifying the significance of the moderated mediation effect of leader incivility by performing a comparative analysis of different types of organizations.

Fourth, organizational phenomena are rarely influenced by a single antecedent in isolation; knowledge-hiding behavior is difficult to predict via its complexity-different antecedent variables, and complex interactions are expected to drive knowledge-hiding in organizations (Moh’d et al., 2021). Future research should focus on both organizational and supervisory aspects to explore the antecedent variables and demonstrate the complex interactions of various moderating effects.

Finally, while our research sheds various meaningful insights into the issues leading to knowledge hiding, the cross-sectional nature of our data is regarded as tenuous when making inferences and conclusions. We emphasize that perceptions of exploitative leadership, leader incivility, and psychological distress can change over time. This eventually leads to changes in knowledge hiding. Therefore, we proposed a longitudinal study to measure these variables and describe any changes over time.

## Data availability statement

The raw data supporting the conclusions of this article will be made available by the authors, without undue reservation.

## Ethics statement

The studies involving humans were approved by Gachon university in Republic of Korea. The studies were conducted in accordance with the local legislation and institutional requirements. The participants provided their written informed consent to participate in this study. Written informed consent was obtained from the individual(s) for the publication of any potentially identifiable images or data included in this article.

## Author contributions

XJ: Data curation, Methodology, Writing – original draft. SJ: Conceptualization, Formal analysis, Writing – review & editing. CQ: Conceptualization, Writing – review & editing.
